# Prevalence of Getah Virus in Mammals in East and Southeast Asia: A Systematic Review and Meta‐Analysis

**DOI:** 10.1155/tbed/8892716

**Published:** 2026-06-19

**Authors:** Zhen Lei, Tong Zhou, Mengda Du, Dongyang Li, Baishi Lei, Kuan Zhao, Yunhang Zhang, Wanzhe Yuan, Jie Tong, Jiangwei Song, Wuchao Zhang

**Affiliations:** ^1^ College of Veterinary Medicine, Hebei Agricultural University, Baoding, 071000, China, hebau.edu.cn; ^2^ Beijing Key Laboratory for Prevention and Control of Infectious Diseases in Livestock and Poultry, Institute of Animal Husbandry and Veterinary Medicine, Beijing Academy of Agriculture and Forestry Sciences, Beijing, 100000, China, baafs.net.cn; ^3^ School of Public Health, Nanchang University, Nanchang, 330000, China, ncu.edu.cn; ^4^ College of Marine Life Sciences, Ocean University of China, Qingdao, 266000, China, ouc.edu.cn; ^5^ National Research Center of Engineering and Technology for Veterinary Biologicals, Nanjing, 210014, China; ^6^ College of Life Science, Hebei University, Baoding, 071002, China, hbu.cn

**Keywords:** arbovirus, Getah virus, meta-analysis, prevalence, veterinary epidemiology

## Abstract

Getah virus (GETV) has reemerged as an important mosquito‐borne pathogen affecting livestock health in East and Southeast Asia. To quantify its epidemiological patterns, we conducted a Preferred Reporting Items for Systematic Reviews and Meta‐Analyses (PRISMA)‐compliant meta‐analysis of literature published between 2000 and September 2025. Analysis of 24 observational studies covering swine, equine, bovine, caprine, and wildlife populations using a random‐effects model revealed a pooled mammalian seroprevalence of 26% (95% confidence interval [CI]: 0.17–0.38) and a pooled nucleic acid positivity rate of 2% (95% CI: 0.01–0.05), consistent with broad past exposure but limited detection of active viremia in cross‐sectional surveys. Swine were identified as the main amplifying hosts, particularly in intensive farming regions of eastern and southern China, where herd seropositivity exceeded 60%. Results indicate a significant prevalence increase over the past 25 years. The established mosquito–swine–mosquito transmission cycle confirms GETV has transformed from a sporadic pathogen into a persistent threat within intensive farming environments.

## 1. Introduction

Over the past five decades, it has become clear that arthropod‐vectored viruses (arboviruses) have evolved into a major challenge to global public health. The geographical distribution of these pathogens is expanding, driven by global warming, ecological changes, and intensified international trade, leading to frequent cross‐species transmission events. Among these viruses, members of the *Alphavirus* genus within the *Togaviridae* family have attracted considerable attention due to their broad host spectrum and zoonotic potential [1].

Getah virus (GETV), a mosquito‐borne pathogen, has historically been overlooked or misdiagnosed as other viral infections due to its typically mild clinical symptoms. However, recent epidemiological data indicate that the prevalence of this virus is showing an upward trend in East Asia. The consequences of this phenomenon are twofold, posing a serious health threat and economic burden to domestic animals such as horses, swines, and blue foxes and raising potential public health concerns [2, 3].

GETV was first isolated in 1955 from *Culex* spp. in Malaysia and has since been detected in a wide geographical area, including Japan, China, South Korea, and Mongolia [4, 5]. Although this virus has historically been classified as an Old World alphavirus that causes only mild pathological changes in animals, recent molecular surveillance data have challenged this traditional view [6, 7]. The virus appears to be undergoing rapid evolutionary divergence, which is particularly noticeable in China, where the isolation rate of GETV has risen dramatically since 2000 and has been accompanied by multiple outbreaks of pathogenicity in swine and equine herds [5].

Despite the large number of case reports and local serological survey data that have been accumulated, comprehensive analyses of mammalian GETV prevalence are still lacking. The existing literature shows significant heterogeneity, which is mainly attributed to disparate sampling timelines, geographic fragmentation, host species variability, and inconsistent detection methodologies ranging from serological assays to molecular techniques and viral isolation. This fragmentation of data blurs our understanding of the epidemiological patterns of the virus and hinders the development of evidence‐based prevention and control strategies. Therefore, conducting a meta‐analysis of existing literature to evaluate GETV prevalence in mammals is both scientifically significant and urgently needed.

GETV is an enveloped, positive‐sense single‐stranded RNA (+ssRNA) virus belonging to the genus *Alphavirus* within the family *Togaviridae*. The 3′‐terminal ORF encodes five structural proteins (Capsid, E3, E2, 6K, and E1). Among these, the E1 and E2 glycoproteins form spike structures on the virus surface, serving as the primary antigenic determinants that mediate viral attachment, host cell entry, and the induction of neutralizing antibodies [8, 9].

Phylogenetic analysis reveals that GETV is closely related to Ross River virus (RRV), Chikungunya virus (CHIKV), and Sagiyama virus (SAGV). Current molecular epidemiological studies have classified GETV into four main evolutionary lineages (Group I–IV). Group III and Group IV are currently the main epidemiological lineages in the East Asian region, especially in China and Japan, suggesting that the virus has adapted and selected for the ecological environment of the region [10]. GETV is detected in coinfections with atypical porcine pestivirus (APPV) in cases of reproductive failure, presenting a complex diagnostic challenge [11].

GETV is a typical arbovirus, maintained in nature through an enzootic mosquito–vertebrate–mosquito transmission cycle. Vector competence is at the core of this transmission dynamics; the virus has been isolated from a diverse array of mosquitoes across the *Culex*, *Aedes*, *Anopheles*, and *Armigeres* genera. In East Asia, *Culex tritaeniorhynchus* and *Armigeres subalbatus* are currently recognized as the primary vectors driving viral amplification [12].

The clinical significance of GETV was clearly defined in 1978 when a large animal outbreak occurred in the racehorse population in the Kanto region of Japan. Infected horses exhibited symptoms such as pyrexia (39–41°C), hind limb edema, generalized rash, lymph node enlargement, and compromised athletic performance [13]. In recent years, swine have become critical reservoir hosts, especially in high‐density farming systems in China [14]. Clinical signs of infection in swine include fever, anorexia, and neurological signs such as tremors and hind limb paralysis. Of particular concern is the virus’s impact on reproductive health; GETV can be transmitted transplacentally in pregnant sows, leading to abortion, stillbirth, fetal mummification, and high mortality in neonatal piglets [15].

In addition to equines and swine, GETV also poses a threat to the fur animal farming industry. An outbreak on blue fox farms in Shandong Province, China, led to cases of fever, anorexia, and death, resulting in serious economic losses [10]. From a public health perspective, while no severe human cases have been confirmed, the frequent detection of neutralizing antibodies in both the febrile patients and healthy cohorts indicates that zoonotic exposure is common. Given its proven ability for cross‐species transmission, increased vigilance is required to consider GETV as a potential emerging human pathogen [5].

The geographic distribution of GETV shows clear regional patterns, primarily concentrated in the Asia‐Pacific region. Since 2010, the number of GETV isolates in China has increased dramatically, spanning a wide geographic range from the tropical Hainan Island to the cold climates of Heilongjiang Province and from the eastern seaboard to inland provinces like Xinjiang and Gansu [14, 16]. This expansion may be attributed to climate change driving the northward migration of vector habitats and the trans‐regional transport of livestock.

In response to the current fragmentation of epidemiological data, this study conducted a meta‐analysis of the literature published between 2000 and 2025 with the aim of assessing the prevalence of GETV in mammals. By consolidating these disparate datasets, we aim to clarify the virus’s ecological footprint and fill critical gaps in global surveillance. The insights gained from this research will provide a scientific basis for veterinary authorities to improve immunization strategies, optimize monitoring network layouts, and establish robust barriers against interspecies transmission. These efforts are crucial for safeguarding livestock biosecurity and public health.

## 2. Materials and Methods

### 2.1. Protocol and Registration

This systematic review and meta‐analysis strictly followed the Preferred Reporting Items for Systematic Reviews and Meta‐Analyses (PRISMA) guidelines [17]. The study protocol was registered with the Open Science Framework (OSF; DOI: 10.17605/OSF.IO/2XYM6).

### 2.2. Search Strategy

We conducted a comprehensive literature search in PubMed, Web of Science, ScienceDirect, and CNKI for studies published between January 2000 and September 2025.

The search strategy employed a combination of Medical Subject Headings (MeSH) and free‐text terms using Boolean operators. The specific search strings included variations of the pathogen name, host species, and epidemiological outcomes: (Getah virus OR GETV OR Alphavirus) AND (mammal OR horse OR equine OR swine OR porcine OR cattle OR bovine OR sheep OR goat OR wildlife OR domestic animals OR wild animals) AND (seroprevalence OR prevalence OR serological survey OR serosurveillance OR epidemiology OR cross‐sectional OR outbreak OR surveillance OR infection rate).

To ensure data saturation, the reference lists of retrieved articles were manually screened for additional eligible studies.

### 2.3. Inclusion and Exclusion Criteria

Study eligibility was assessed using the PICO framework (Population, Intervention/Exposure, Comparator, and Outcome) tailored for epidemiological prevalence studies: Population (P): Mammalian populations (including, but not limited to, swine, equines, and bovines). Intervention/exposure (I): natural infection with or exposure to the GETV. Comparator (C): not applicable. As this study aims to estimate the pooled prevalence derived from single‐arm observational surveys, a comparator group is not a prerequisite for inclusion. Outcome (O): prevalence of GETV infection was defined as the proportion of positive cases relative to the total sample size. This encompasses both seroprevalence (the detection of specific antibodies) and virological prevalence (the detection of viral RNA or antigen).

Inclusion criteria studies were included if they met the following criteria: (a) observational studies (e.g., cross‐sectional surveys, cohort studies, or surveillance reports) that reported GETV prevalence data. (b) Studies providing extractable data on both the total sample size and the number of positive cases. (c) Studies utilizing validated diagnostic assays, specifically enzyme‐linked Immunosorbent assay (ELISA), hemagglutination inhibition (HI), virus neutralization test (VNT), RT‐PCR, or virus isolation. (d) Full‐text articles published in English or Chinese.

Exclusion criteria studies were excluded if they met any of the following conditions: (a) reviews, meta‐analyses, conference abstracts, letters to the editor, or editorials. (b) Case reports or case series with small sample sizes (*n* < 30) to minimize sampling error and bias. (c) Experimental infection studies or vaccine efficacy trials. (d) Studies containing insufficient data to calculate the point estimate of prevalence. (e) Duplicate publications or studies utilizing overlapping datasets. In such instances, the article with the most comprehensive data or the largest sample size was retained.

### 2.4. Study Selection Process

All literature records were imported into EndNote software for automatic duplicate removal. Two reviewers independently screened the titles and abstracts against eligibility criteria, followed by a full‐text review of potential candidates. Disagreements, if any, are resolved through negotiation or arbitration by a senior evaluator. The full selection workflow is detailed in the PRISMA flow diagram.

### 2.5. Data Extraction

Data extraction was carried out independently by two researchers using pretested standardized forms. Captured variables included study metadata (author, year, and region), population demographics (host species and sampling source), methodological details (assay type and sample matrix), and quantitative outcomes (sample size, positive cases, and prevalence). If key information is missing or unavailable, the study in question will be excluded.

### 2.6. Quality Assessment

The risk of bias was evaluated using the Joanna Briggs Institute (JBI) Critical Appraisal Checklist for Prevalence Studies. The evaluation process was conducted independently by two individuals, and any discrepancies were resolved through discussion. Notably, Item 9 (response rate) was adapted to fit veterinary surveillance contexts, rather than participant response; this criterion assessed data completeness (i.e., whether all collected samples were tested and included), with unexplained exclusions scored as “No.”

### 2.7. Statistical Analysis

All analyses were executed in Python 3 (Pandas, Statsmodels, and Scipy). To ensure statistical independence, studies involving multiple host species were separated into independent analytical units. For zero‐event studies, a continuity correction of 0.5 was applied to avoid computational errors.

Given the high variability between studies, a logit transformation was applied to the original proportions to ensure that the confidence intervals (CIs) remained within the [0, 1] range. Pooled prevalence was estimated using a DerSimonian–Laird random‐effects model, anticipating significant heterogeneity (*I* ^2^ >50%) [18, 19]. Heterogeneity was quantified using the Cochran’s *Q* test and *I* ^2^ statistic, and sources of heterogeneity were explored by subgroup analyses (by assay) and univariate meta‐regression (by host and year). Publication bias was assessed using funnel plots and Egger’s test (*p* < 0.05), while result robustness was validated through leave‐one‐out sensitivity analysis [20].

## 3. Results

### 3.1. Literature Selection and Characteristics of Included Studies

**Figure 1:**
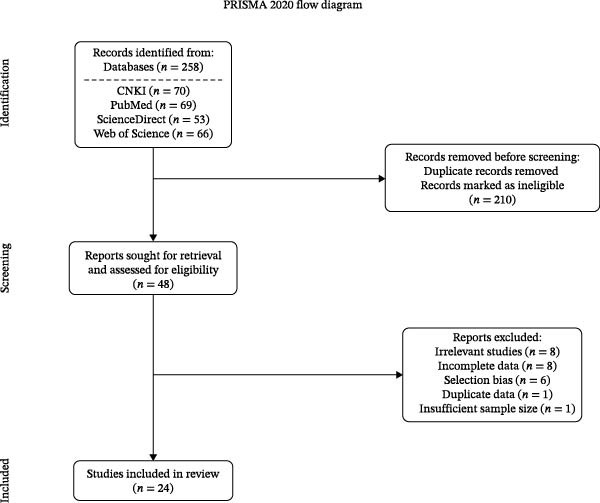
PRISMA 2020 flow diagram illustrating the literature search and selection process. A total of 258 records were initially identified from CNKI, PubMed, ScienceDirect, and Web of Science. After removing duplicates and screening titles/abstracts (*n* = 210), 48 full‐text articles were assessed for eligibility. Twenty‐four studies were excluded due to reasons such as insufficient sample size, duplicate data, high risk of bias, or incomplete data. Ultimately, 24 studies met the inclusion criteria and were included in the meta‐analysis.

The literature screening process strictly followed the PRISMA 2020 guidelines as shown in Figure 1. An initial search of several databases (CNKI, PubMed, ScienceDirect, and Web of Science) yielded 258 records. After de‐duplication and initial screening, we assessed the eligibility of 48 full‐text articles. Ultimately, 24 studies met all inclusion criteria and were included in the final quantitative synthesis analysis.

These included studies span a 15‐year period from 2010 to 2025 and primarily originated from China, Japan [3, 21–24], and Thailand [25]. The dataset encompasses a diverse range of host species, including swine [22, 25–32], equine [3, 21, 28, 30, 33, 34], bovine [28, 30, 31, 35], caprine [28, 30], and wild boars [23, 24] (Table 1).

**Table 1:** Studies included in the meta‐analysis of Getah virus prevalence in mammals.

Authors	Publication years	States	Method_Group	Host_Group	Sample size	Number of positive	Positive rate (%)
Wu et al.	2024	China	Nucleic acid	Swine	2512	84	3.34
Chang et al.	2025	China	Nucleic acid	Swine	374	5	1.34
Shen et al.	2024	China	Nucleic acid	Swine	2110	—	5.86
Liang et al.	2010	China	Antibody	Swine	147	24	16.33
Qiu et al.	2022	China	Antibody	Equine	294	227	77.2
Liang et al.	2010	China	Antibody	Humans	300	50	16.67
Bannai et al.	2016	Japan	Antibody	Racehorses	1992	30	1.5
Li et al.	2025	China	Antibody	Swine	150	69	46
Rattanatumhi et al.	2022	Thailand	Antibody	Swine	1188	275	23.1
Qiu et al.	2022	China	Antibody	Horse/Equine	646	334	51.7
Bannai et al.	2015	Japan	Antibody	Racehorses	1950	64	3.3
You et al.	2024	China	Antibody	Swine	986	347	35.2
Cao et al.	2022	China	Nucleic acid	Swine (*n* = 1106) Horses (*n* = 597) Bovines (*n* = 667) Sheep(*n* = 477)	2847	Swine (*n* = 11) Horses (*n* = 6) Bovines (*n* = 1) Sheep (*n* = 0)	0.6
Liu et al.	2019	China	Nucleic acid	Cattle	48	3	6.25
Bannai et al.	2017	Japan	Antibody	Swine	918	148	16.12
Shen et al.	2025	China	Nucleic acid	Swine	1382	57	4.12
Kuwata et al.	2018	Japan	Antibody	Wild boar	1048	168	16
Shi et al.	2022	China	Nucleic acid	Thoroughbred horse (200) Local horse (210) Goat (100) Sheep (120) Cattle (110) Swine (128)	868	Thoroughbred horse (2) Local horse (1) Goat (0) Sheep (0) Cattle (0) Pigs (1)	0.46
Zhong et al.	2024	China	Antibody	Horse/Equine	182	52	28.6
Liu et al.	2023	China	Nucleic acid and antibody	Bovine	534 (Antibody) 1300 (Nucleic acid)	108 (Antibody) 3 (Nucleic acid)	20.25 (Antibody) 0.23 (Nucleic acid)
Sugiyama et al.	2009	Japan	Antibody	Wild boars	90	43	47.8
Li et al.	2018	China	Antibody	Dairy cattle (*n* = 15) Pig (*n* = 85) Beef cattle (*n* = 32)	132	Dairy cattle (*n* = 2) Pig (*n* = 39) Beef cattle (*n* = 23)	Dairy cattle (13) Pig (46) Beef cattle (72)
Sun et al.	2022	China	Antibody	Swine	133	50	37.59
Lan et al.	2025	China	Antibody	Swine	2102	1239	58.94

### 3.2. Characteristics of Included Studies

The 24 included studies were predominantly sourced from China, which covers a wide area from the hinterland to the coastal provinces. Complementary longitudinal data from Japan and Thailand provided critical insights into GETV dynamics in racehorses and wild boars.

The included studies were classified into two main categories based on the objectives of the assays, antibody assays (serological investigations, *n* = 17) and nucleic acid assays (molecular biological investigations, *n* = 10). Because several studies reported both serological and molecular findings, the total number of assay‐specific analytical units exceeds the 24 included studies. These serological surveys utilized assays, including ELISA (encompassing indirect, recombinant rE2‐, and rCap‐ELISA variants), VNT, HI, and luciferase Immunoprecipitation Systems (LIPS). Some studies employed molecular biology techniques, primarily reverse transcription polymerase chain reaction (RT‐PCR) and real‐time quantitative PCR (RT‐qPCR).

### 3.3. Risk of Bias Assessment

Methodological quality was evaluated using the JBI Critical Appraisal Checklist, and the results are summarized in Figure S1. The primary source of bias was the sampling strategy (Item Q2). Due to the objective limitations of practical work in veterinary epidemiology, research is often forced to use convenience sampling rather than probability sampling. Although this introduces a potential selectivity bias, the overall quality of the included studies was assessed to be moderate to high and still of high analytical value for characterizing regional epidemiological trends.

### 3.4. Meta‐Analysis Results

**Figure 2:**
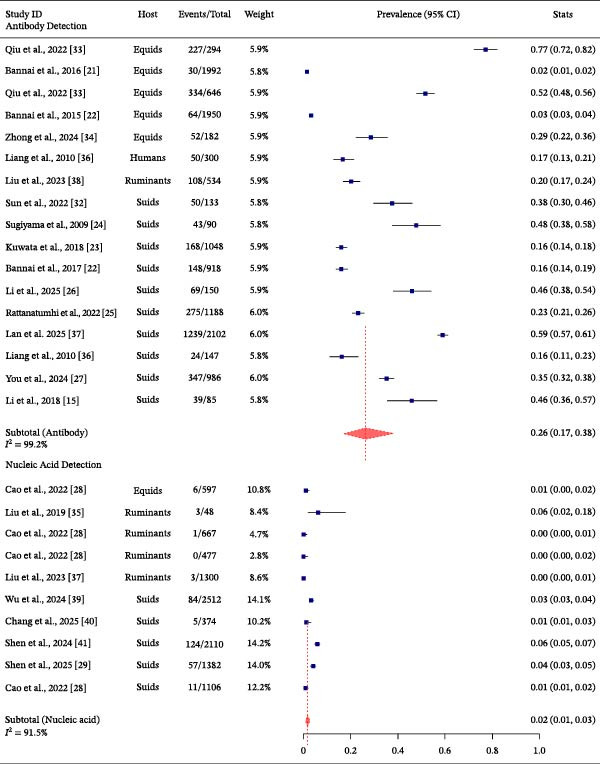
Forest plot detailing the pooled prevalence of Getah virus (GETV) infection in mammals, stratified by detection method. The meta‐analysis is categorized into two subgroups based on diagnostic assays: Antibody Detection (indicating historical exposure) and Nucleic Acid Detection (indicating active viral infection). For each included study, the blue square represents the point estimate of the prevalence, with the horizontal line extending to the 95% confidence interval (CI). The red diamonds at the bottom of each section indicate the pooled subtotal prevalence calculated via a random‐effects model. The *I* ^2^ statistic denotes the degree of between‐study heterogeneity within each subgroup.

As shown in the forest plot (Figure 2), there were significant differences in the estimated prevalence rates obtained using the different detection methods. The antibody detection subgroup (*n* = 17) revealed a high pooled seroprevalence of 26% (95% CI: 0.17–0.38), indicating extensive subclinical circulation and historical exposure across East Asian mammal populations.

Equine herds in Xinjiang show very high seropositivity rates. Qiu et al. [33] reported rates of 77.2% (95% CI: 72.2%–81.9%) in 2020, stabilizing at 51.7% in 2021. These data reflect the intense natural infection pressure in the region, which is likely primarily due to its vast pastoral landscape and high density of vectors, facilitating efficient mosquito‐to‐horse transmission. Similarly, swine in southeastern China (Jiangxi and Fujian) showed high endemicity (58.94%), while recent surveillance in Hebei reported 46% seropositivity [26, 37]. These findings suggest that GETV has established stable endemic transmission in areas characterized by intensive swine farming and a humid climate. In contrast, historical data from Japanese racecourses indicate significantly lower incidence rates (1.5%–3.3%) [3, 21].

The pooled nucleic acid positivity rate from 10 molecular biology studies was significantly lower, at only 2% (95% CI: 0.01–0.03). This contrast between 26% and 2% is consistent with the dynamics of arboviruses, whereby immunocompetent hosts usually have a short viraemia period, resulting in an inherently low probability of capturing an active viral detoxification period in cross‐sectional surveys. Notably, while cattle in specific foci (e.g., Yunnan) exhibited seropositivity up to 20%, viral RNA detection remained negligible [38].

However, it is important to note that this low overall data mask the high detection rates observed during acute outbreaks. For example, during the 2024 epidemic in Henan Province, the PCR detection rate in aborted fetuses and sick piglets was extremely high, demonstrating that under specific spatiotemporal conditions, the viral load can rapidly reach a peak [7].

The distribution of the sample size in relation to prevalence (Figure S2) further supports these findings. Suids and equids are not only the main targets for surveillance but also seem to be the most important susceptible and amplifying hosts for GETV.

### 3.5. Host Infection Dynamics and Cross‐Species Transmission Risks

**Figure 3:**
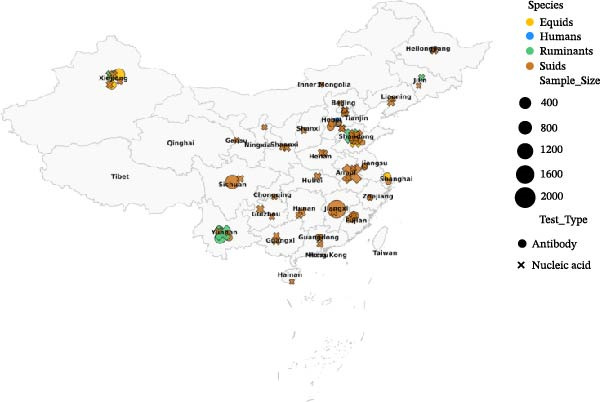
Geographical distribution of samples by province in China. The map details the sampling locations and associated data attributes. Colors distinguish the species sampled: humans, equids, ruminants, and suids. Shapes identify the type of assay circles for antibody assays and crosses for nucleic acid assays. Bubble size is proportional to the sample size at each sampling point.

Swine occupy a central position in the epidemiological chain of GETV (Figure 3). Large‐scale serological surveillance in Jiangxi (2018–2024) showed antibody positivity rates as high as 63.36% in some swine herds [37]. In terms of clinical, the phenotypic spectrum of GETV in swine is expanding; in addition to classical reproductive disorders, recent isolates have been associated with severe intestinal and neurological syndromes (diarrhea, ataxia, and tremors) and subcutaneous edema [42].

Equids are highly susceptible to GETV and exhibit characteristic clinical symptoms, including fever, rash, and hindlimb edema, making them ideal sentinel animals for monitoring viral activity [4, 43]. In China, equine infections are mainly disseminated or characterized by localized epidemics. Age‐stratified data from Shanghai showed evidence of a cumulative immune effect, with the seropositivity rate peaking in horses older than 15 years (41.7%). Moreover, the prevalence rate was significantly higher in autumn (45.1%) than in spring (12.1%), a clear seasonal pattern that is highly consistent with the vector extinction curve, further confirming the dominance of mosquito‐borne transmission [44].

Although the detection rate of bovine nucleic acid is extremely low nationwide, there is a significant exception in the China–Myanmar border region (Honghe Prefecture, Yunnan Province). Here, bovine seroprevalence reached 20.25%, and the first bovine‐derived strain (YN2305) was successfully isolated [38]. This finding warrants further investigation of local ecological conditions, including vector abundance, seasonal transmission intensity, and the role of *Culex tritaeniorhynchus* in this border corridor.

### 3.6. Regional Distribution Characteristics in China

As shown in Figure 3, southwest China is the core epidemic area with a long history of epidemics and the highest host diversity. Several GETV strains isolated during investigations in Yuxi and Honghe Prefecture are phylogenetically closely related to strains from Southeast Asian countries such as Myanmar and Laos [38]. The region’s permissive climatological conditions, characterized by high humidity and temperature, sustain the perennial activity of competent vectors, primarily *Culex tritaeniorhynchus*.

In contrast, central and eastern China have become major locations for recent pathogenic outbreaks and associated economic losses. Henan Province is a core region for swine production in China, and the GETV outbreak that occurred in the province in 2024 affected 21 counties, demonstrating its extremely high transmission efficiency [7]. Henan’s dense transport network and frequent swine transfers make it a key hub for the spread of the virus. In East China, the antibody‐positive rate in swine populations in provinces such as Jiangxi and Fujian has consistently remained at a high level of 35% to 65% [37]. In Shanghai, infections are mainly concentrated in the racecourse environment, whose water network system provides an ideal breeding ground for *Culex* mosquito populations [44].

Although no geographic restriction was applied during the literature search, all studies eligible for the final synthesis were from East and Southeast Asia; accordingly, the regional distribution patterns described here reflect the currently available evidence from this geographic area.

### 3.7. Temporal Trends

**Figure 4:**
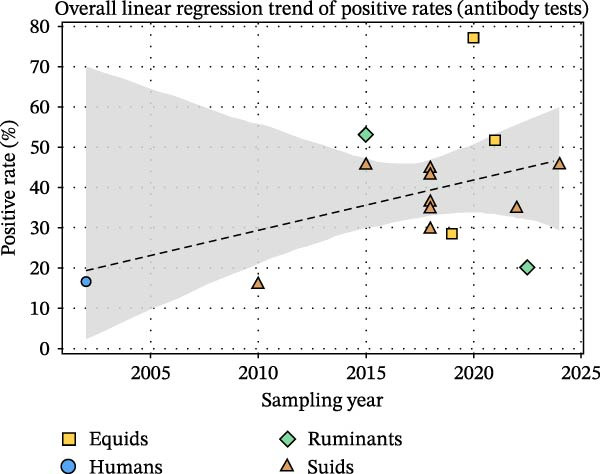
Temporal trends in seroprevalence across all species. Linear regression analysis shows the overall statistical trend in antibody positivity rates over the multiyear sampling period.

Temporal regression analysis (2000–2024) revealed a statistically significant long‐term increasing trend in seroprevalence among mammal populations (Figure 4). This pattern may reflect more than one process. A biological expansion of GETV circulation is plausible, but the observed increase is also likely influenced by improved surveillance intensity and the wider use of ELISA‐ and PCR‐based diagnostics in recent years. In the early phase (2000–2010), the available data were relatively sparse and mainly derived from wild boar monitoring in Japan and sporadic surveys in China, which likely limited case detection.

The 2014–2015 GETV outbreaks in Japan became a landmark epidemiological event; although the point prevalence at the time was moderate (~3.3%), it significantly raised awareness of the disease [3]. Meanwhile, a survey conducted in Yunnan in 2015 revealed a high background infection rate (48.5%), confirming that the virus has established a stable transmission cycle in natural reservoir hosts [31]. The prevalence rate peaked in the past 5 years. Surveys conducted on horses in Xinjiang (2020/2021), swine in Sichuan (2022), and swine in Jiangxi/Fujian (2023–2024) consistently reported extremely high prevalence rates, ranging from 35% to 77% [27, 33, 37].

This sharp upward trend may be the result of a synergy of factors. Global warming has extended the active season of vector mosquitoes such as *Culex tritaeniorhynchus* and Culex *quinquefasciatus* and expanded their geographical distribution northward (e.g., extending to Hebei and Jilin provinces) [45]. Intensification of livestock systems increases host densities and thus facilitates rapid in‐group amplification of the virus after introduction [37]. Furthermore, the widespread application of diagnostic techniques (especially ELISA and qPCR) has enhanced passive and active surveillance capabilities, thereby improving the detection rate of subclinical infections.

### 3.8. Sensitivity Analysis and Publication Bias

**Figure 5:**
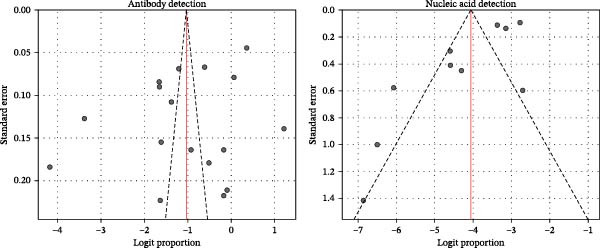
Funnel plot for assessing publication bias. Funnel plots were drawn separately for each of the two main subgroups. The horizontal axis (*x*‐axis) is the logit‐transformed prevalence and the vertical axis (*y*‐axis) is the standard error. The solid red vertical line represents the combined prevalence estimate. The scatter is symmetrically distributed around the red line and is mainly concentrated in the inverted triangle region, suggesting that there was minimal publication bias in the study.

The funnel plot (Figure 5) revealed different asymmetry patterns among subgroups. In prevalence meta‐analysis, especially under substantial heterogeneity, funnel plot asymmetry should not be interpreted as publication bias alone. The relative symmetry of the serological dataset is compatible with more stable prevalence estimates, whereas the asymmetry in the molecular dataset may also reflect outbreak‐focused sampling, true between‐study differences, and methodological variation in addition to possible small study effects.

**Figure 6:**
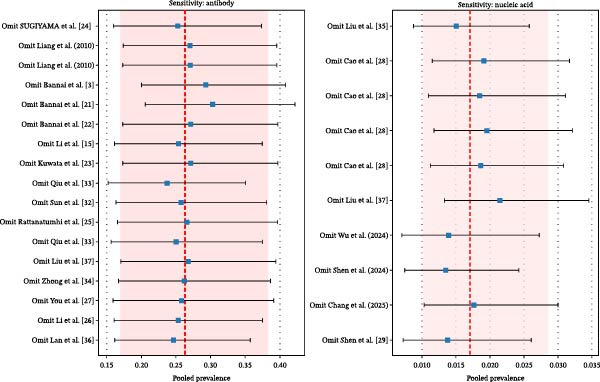
Sensitivity analysis plot for assessing the robustness of the pooled estimate. Each row shows the recalculated pooled prevalence (blue square) and its 95% confidence interval after excluding the specific study labeled on the left. The red dashed line represents the baseline pooled prevalence, including all original studies. The red shaded area indicates the 95% confidence interval range of the original pooled estimate.

We further verified the robustness of the results through leave‐one‐out sensitivity analysis (Figure 6). For the antibody and nucleic acid subgroups, the exclusion of any single study, including those with the largest sample sizes or highest prevalence rates, did not result in statistically significant deviations from the recalculated combined prevalence rates. Crucially, all revaluations remained within the 95% CI of the original aggregate estimate.

## 4. Discussion

Historically, the GETV has often received limited attention in veterinary epidemiology compared with other mosquito‐borne viruses. In this meta‐analysis of 24 studies published between 2000 and 2025, we found evidence of substantial mammalian exposure to GETV in East and Southeast Asia, particularly in intensive livestock production systems in China. However, because the included studies were concentrated in China, Japan, and Thailand, any broader inference about the global GETV risk should be made cautiously.

The main finding of this meta‐analysis was the marked difference between serological and molecular indicators. The pooled seroprevalence was 26%, whereas the pooled molecular positivity rate was 2%. This pattern is biologically plausible because, in hosts such as swine and equine, the viremic window is brief, often lasting only a few days. Unless sampling coincides with the acute phase of infection, PCR‐based surveillance is unlikely to detect many positive animals.

By contrast, postinfection antibodies may persist substantially longer than detectable viremia [7]. Thus, the observed 26% seroprevalence effectively documents the historical trajectory of high‐frequency, subclinical transmission of the virus in relevant animal populations over the past several years. This finding serves as an important warning: current clinical reports only reveal the tip of the iceberg regarding the true epidemiological burden. Clearly, GETV has established a stable transmission cycle in the environment. At the same time, differences in serological assay design should be considered when interpreting these pooled estimates. Because E2 is a major surface antigen and a key target of neutralizing antibodies, E2‐based assays appear particularly suitable for longer‐term serological screening; however, more direct comparative studies are needed before any single platform can be considered a definitive field standard [46].

Undoubtedly, swine have become the most important natural amplification host for GETV in East Asia. Monitoring data from Jiangxi and Fujian provinces show that the seropositivity rate in some swine populations exceeds 60% [37]. This high exposure is directly related to the high‐density intensive farming model prevalent in China. In the confined environment of a swine farm, where the genetic background of the animals is uniform, once a virus‐carrying vector breaches the biosecurity barrier, the virus can exploit the host’s ability to produce high‐titer viremia, leading to a rapid, exponential spread. Even more worrying is that the GIII subtype variant has evolved the ability to cause severe mortality in newborn piglets [7]. Thus, swine are not only amplifiers of viruses but also evolutionary testing grounds for viruses to enhance pathogenicity.

In contrast, the epidemiological landscape of the horse population exhibits a significant binary division, which directly depends on the intensity of human intervention. In the vast pastoral areas of Xinjiang, the seroprevalence rate of horses under rough‐grazing systems is as high as 77.2% [33]. This data accurately reflects the density of pathogens in the environment and illustrates the immense infectious pressure that equids face in their natural habitat. At strictly regulated Japanese racecourses, thanks to systematic vaccination and vector control, the infection rate has been successfully suppressed to a negligible level [21, 47]. This dramatic contrast precisely confirms the unique epidemiological value of horses as sentinel animals for monitoring the environmental viral load.

Data from Liu et al. [38] study along the China–Burma border provide key evidence for defining the role of cattle in the virus transmission chain. Although the cattle in this region showed a seropositivity rate of ~20%, their nucleic acid positivity rate was as low as 0.23%. This suggests that it is extremely difficult for cattle to develop a high‐titer viraemia sufficient to maintain a “mosquito–cattle–mosquito” cycle after infection. From a disease transmission dynamics perspective, cattle tend to act as terminal hosts; they develop an immune barrier by passively receiving mosquito bites, rather than serving as the primary reservoir for the maintenance and spread of the virus.

Regression analyses suggest an upward trajectory of GETV seroprevalence overtime, but this pattern should not be attributed to biological expansion alone. Climate‐related changes, shifts in vector ecology, livestock intensification, and animal movement are all plausible explanations, yet none of these drivers was tested directly in the present meta‐analysis. The survival isotherm of *Culex tritaeniorhynchus*, the main vector mosquito, is steadily advancing toward higher latitudes [45]. The synergistic effect of warmer temperatures, i.e., the lengthening of the vector season and the shortening of the extrinsic incubation period of the virus in mosquitoes, opens up ecological corridors for the expansion of the virus into temperate regions. The increasing availability of ELISA‐ and qPCR‐based surveillance may also improve case finding. The present study can identify the temporal pattern, but it cannot distinguish the relative contribution of these mechanisms.

The geographical distribution map (Figure 3) identifies a unique epidemic source in central China, centered in Henan Province. In stark contrast to Yunnan’s epidemic pattern, which is driven by natural biodiversity, Henan’s highly endemic epidemics exhibit distinct anthropogenic aggregation characteristics. As a national hub for live swine transportation, Henan is a crucial node in the movement of breeding swine and market swine. The large‐scale outbreaks reported earlier were likely facilitated by this modern logistic network [7]. This high‐frequency cross‐regional flow not only breaks down natural geographical barriers but also provides a breeding ground for genetic recombination of strains from different sources, thus accelerating adaptive evolution of viruses [29, 48].

The conclusions of this study are still limited by the inherent methodological flaws in the original data. Most of the included studies used convenience sampling methods, with some samples sourced from animals exhibiting clinical symptoms or from specific slaughterhouses. This method introduces a selection bias and may, to some extent, overestimate the actual overall prevalence rate.

Furthermore, the low nucleic acid positivity rates observed during nonepidemic periods may create a false impression that the disease has been eliminated, masking potential environmental risks. However, even considering these biases, the substantial baseline of infections revealed by serological data still provides strong evidence, confirming the widespread asymptomatic transmission of GETV.

## Author Contributions

**Zhen Lei:** conceptualization, methodology, software, formal analysis, investigation, data curation, writing – original draft, visualization. **Tong Zhou and Mengda Du:** methodology, validation, investigation, data curation, writing – review and editing. **Dongyang Li, Baishi Lei, Kuan Zhao, Yunhang Zhang, Wanzhe Yuan, Jie Tong, Jiangwei Song, and Wuchao Zhang:** validation, resources, supervision, project administration, funding acquisition.

## Acknowledgments

During the preparation of this work, the authors used Gemini (Google) in order to improve the readability and language quality of the manuscript.

## Funding

This project was supported by the BAAFS Foundation for Excellent Young Scientists (Grant YXQN202302‐C) and the National Natural Science Foundation of China (Grant 32372980).

## Disclosure

All authors have read and agreed to the published version of the manuscript. After using Gemini (Google), the authors reviewed and edited the content as needed and takes full responsibility for the content of the publication.

## Ethics Statement

This study is a systematic review and meta‐analysis based exclusively on data extracted from previously published literature and public databases. It does not involve any primary data collection, animal experiments, or human participants performed by the authors of this study. Therefore, ethical approval from an Institutional Animal Care and Use Committee (IACUC) or Institutional Review Board (IRB) was not required.

## Conflicts of Interest

The authors declare no conflicts of interest.

## Supporting Information

Additional supporting information can be found online in the Supporting Information section.

## Supporting information


**Supporting Information** Figure S1: Risk of bias assessment and summary for the included studies. The assessment is based on the Joanna Briggs Institute (JBI) Critical Appraisal Checklist. Section (a) details the risk of bias for each independent study across nine entries (Q1–Q9), and section (b) Provides a stacked bar chart summarizing the proportion of studies rated as low, high, or unclear risk for each checklist item. Figure S2: Rectangular tree map illustrating the distribution of sample sizes and prevalence. This visualization employs a hierarchical structure, categorized by detection method and host species, to present the distribution characteristics of the data intuitively.

## Data Availability

The data that support the findings of this study are openly available in the Open Science Framework (OSF) at https://doi.org/10.17605/OSF.IO/2XYM6.
