# Supplementary material for: Prevalence of Getah Virus in Mammals in East and Southeast Asia: A Systematic Review and Meta‐Analysis
Source: Transbound Emerg Dis. 2026 Jun 19;2026:8892716. doi: 10.1155/tbed/8892716 (PMC13282556; doi:10.1155/tbed/8892716)
Supplement: Supplementary file 1 — Supporting Information Figure S1: Risk of bias assessment and summary for the included studies. The assessment is based on the Joanna Briggs Institute (JBI) Critical Appraisal Checklist. Section (a) details the risk of bias for each independent study across nine entries (Q1–Q9), and section (b) Provides a stacked bar chart summarizing the proportion of studies rated as low, high, or unclear risk for each checklist item. Figure S2: Rectangular tree map illustrating the distribution of sample sizes and prevalence. This visualization employs a hierarchical structure, categorized by detection method and host species, to present the distribution characteristics of the data intuitively. [file TBED-2026-8892716-s001.pdf]

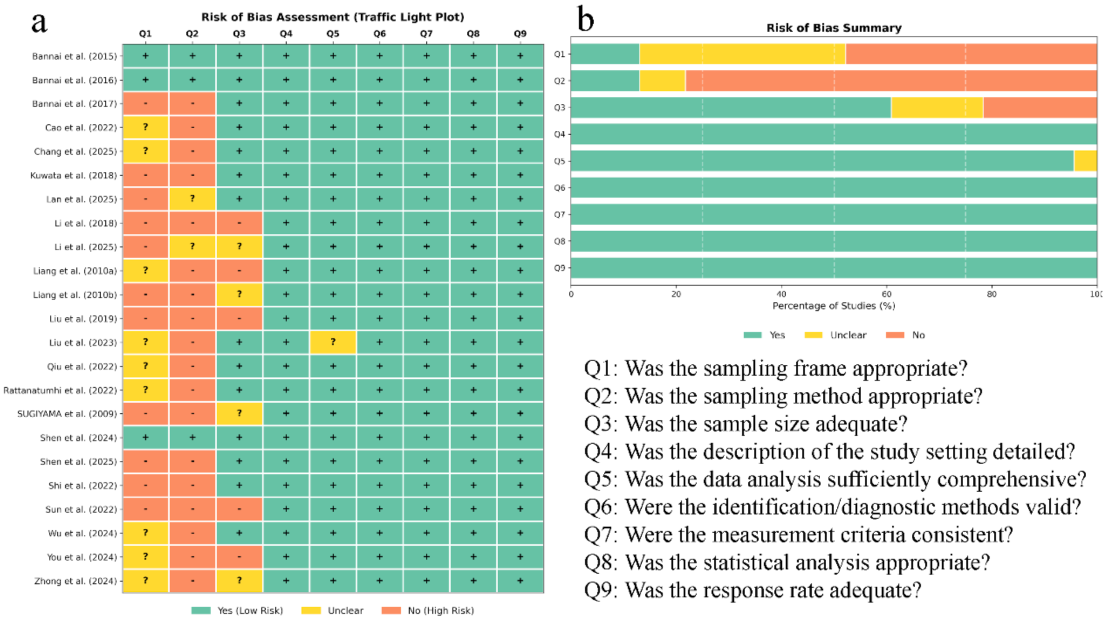

Figure S1. Risk of bias assessment for each included study and Summary of bias risk. Risk of bias assessment for each included study (a) This assessment was based on the Critical Appraisal Checklist for Studies Reporting Prevalence Data published by the Joanna Briggs Institute (JBI). Each row in the figure represents an independent study, and each column corresponds to nine assessment entries (Q1-Q9) in the checklist. Summary of bias risk (b). The stacked bar chart shows the proportion of studies rated as low risk (green), high risk (red), or unclear risk (yellow) for each of the nine items in the JBI checklist.

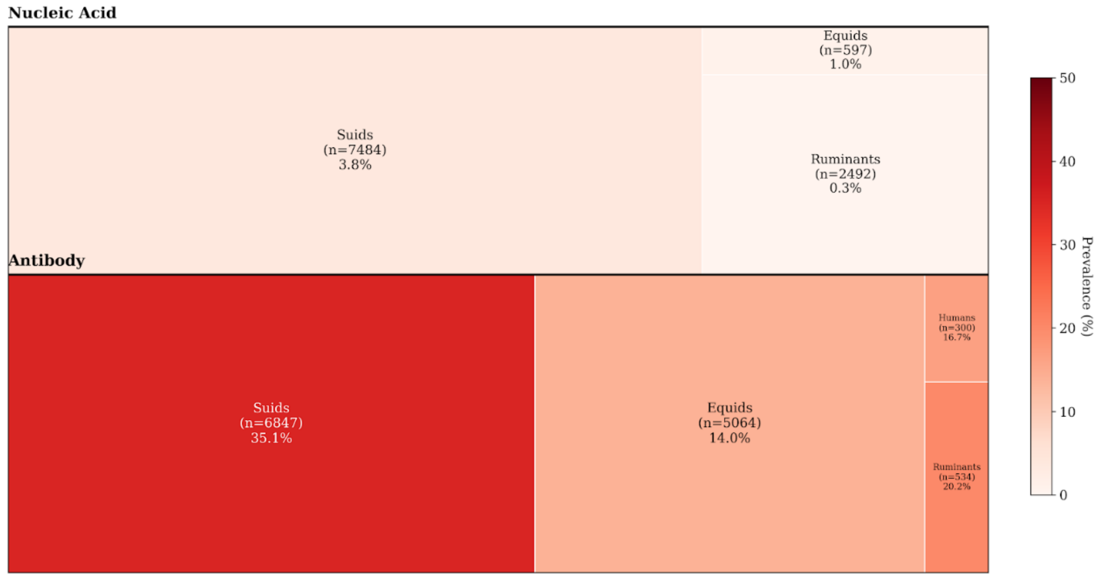

Figure S2. Rectangular tree map showing the distribution of sample size and prevalence across different detection methods and host species. This visualization uses a hierarchical structure, categorized by detection method and host species, to intuitively present the distribution characteristics of the data.
